# Angiotensin II receptor blocker LCZ696 attenuates cardiac remodeling through the inhibition of the ERK signaling pathway in mice with pregnancy-associated cardiomyopathy

**DOI:** 10.1186/s13578-019-0348-1

**Published:** 2019-10-21

**Authors:** Yi Wang, Zhiheng Guo, Yongmei Gao, Ping Liang, Yanhong Shan, Jin He

**Affiliations:** Department of Obstetrics, The First Hospital of Jilin University, No. 71, Xinmin Street, Changchun, 130021 Jilin People’s Republic of China

**Keywords:** Angiotensin II, LCZ696, Extracellular signal-regulated kinase, Pregnancy-associated cardiomyopathy, Cardiac remodeling

## Abstract

Pregnancy-associated cardiomyopathy (PAH) represents a pregnancy-associated myocardial disease that is characterized by the progression of heart failure due to marked left ventricular systolic dysfunction. Compelling evidence has highlighted the potential of angiotensin (Ang) receptor inhibitors as therapeutic targets in PAH treatment. The present study aims to elucidate the molecular mechanisms underlying Ang II receptor inhibitor LCZ696 treatment in PAH. Initially, a PAH mouse model was induced, followed by intraperitoneal injection of LCZ696. Subsequently, cardiomyocytes and fibroblasts were isolated, cultured, and treated with Ang II and LCZ696, followed by detection of the total survival rate, cardiac injury, cardiac fibrosis and apoptosis. Moreover, in order to quantify the cardiac hypertrophy and fibrosis degree of cardiac fibroblasts, the expression levels of markers of cardiac hypertrophy (ANP, βMHC and TIMP2) and markers of fibrosis (collagen I, collagen III and TGF-β) were evaluated. Furthermore, the potential effect of LCZ696 on the extracellular signal-regulated kinase (ERK) signaling pathway was examined. The acquired findings revealed that LCZ696 increased the total survival rate of PAH mice, but decreased cardiac injury, cardiac fibrosis, and apoptosis in vitro. LCZ696 attenuated cardiac injury induced by Ang II through the inhibition the expression of markers of cardiac hypertrophy, fibrosis and apoptosis by inhibiting ERK phosphorylation in vivo and in vitro. Altogether, LCZ676 could potentially alleviate cardiac remodeling in mice with PAH via blockade of the ERK signaling pathway activation. Our findings suggest that LCZ696 could be a potential target for PAH therapy.

## Introduction

Cardiovascular diseases are predominantly known as complications that occur in the prenatal period worldwide, and there are a growing number of patients diagnosed with cardiac disorders during pregnancy [1]. During pregnancy, the physiological changes occurred in the cardiovascular system, including elevated blood pressure, stroke volumes and heart rate could accelerate the initiation of diseases in patients with inherited cardiomyopathy or even those suffering from a mutation that is closely related to cardiomyopathy [2]. Moreover, incessant high expression of angiotensin II (Ang II) has been reported to be correlated with elevated susceptibility to dilated cardiomyopathy and higher mortality of female mice [3]. Besides, Ang II administration has been employed in pregnant hypertensive rats to mimic the augmented cardiovascular stress that is associated to preeclampsia and it was discovered to have a coherent influence on the maternal cardiovascular system and fetal development [4]. Consequently, the inhibitors of Ang II receptor may represent a promising target in the treatment of pregnancy-associated cardiomyopathy (PAH).

LCZ696, also known as sacubitril/valsartan, is identified as a novel dual-acting angiotensin receptor-neprilysin inhibitor (ARNi) and has been extensively reported to approve for the treatment of pediatric heart failure patients with reduced systemic left ventricular systolic function [5]. LCZ696 is recognized as a first-class inhibitor of Ang II receptor, which can completely lessen blood pressure and even aid the treatment of hypertension and cardiovascular diseases [6]. Further work has noted that the activation of the extracellular signal-regulated kinase (ERK) signaling pathway has been proven to accelerate Ang II-induced cardiomyocyte hypertrophy [7]. ERK represents a mitogen activated-protein kinase (MAPK) and participates in the vascular smooth muscle cell growth which was reported to be an assuring therapeutic target for hypertension prevention [8]. More importantly, the protective effect of Asiatic acid against cardiac hypertrophy is attained through the following inhibition of the ERK signaling pathway in vivo and in vitro [9]. Moreover, ERK1/2 is able to be activated by Ang II in vascular smooth muscle cells to suppress insulin receptor substrate-1 tyrosine phosphorylation [10]. The information mentioned previously has led the study to propose a hypothesis that LCZ696 could be implicated in PAH involving the regulation of the ERK signaling pathway. Therefore, this study developed a mouse PAH model through the intraperitoneal injection of LCZ696 and to determine the expected effect of LCZ696 on cardiac hypertrophy, fibrosis, and apoptosis by the regulation of the ERK signaling pathway through application of in vivo and in vitro experiments.

## Materials and methods

### Ethics statement

All animal experimental procedures were approved by the ethics committee of The First Hospital of Jilin University. Extensive efforts were made to ensure minimal suffering of the animals used during the study.

### Cell culture and grouping

The neonatal mouse cardiomyocytes and fibroblasts were separated from the neonatal C57BL/6J mice aged 1–2 days. After enzymatic disassociation, those cells were cultured in a mixture of 4.5 g/L Dulbecco’s modified Eagle’s medium (DMEM; Gibco BRL/Invitrogen, Carlsbad, CA, USA) supplemented with 10% fetal bovine serum (FBS; 10100147, Gibco BRL/Invitrogen, Carlsbad, CA, USA) and high-glucose-M199 medium (Sigma-Aldrich Chemical Company, St Louis MO, USA) at a ratio of 4: 1. The cells were cultured in 4.5 g/L high-glucose DMEM (Gibco BRL/Invitrogen, Carlsbad, CA, USA) and then incubated at 37 °C with 5% CO_2_. Ang II (HY-13948, MCE) and LCZ696 (HY-18204A, MCE) were dissolved in dimethyl sulfoxide (DMSO). When the cardiomyocytes and fibroblasts reached 50% confluence, they were treated with Ang II (100 nM final concentration) for 60 h and LCZ696 (0.03/0.1/0.3/1.0 μmol/L) for 3 h [11]. Moreover, a lentivirus packaging system pSIH1-H1-copGFP (lentivirus short hairpin RNA [shRNA] fluorescent expression vector) was constructed. ERK shRNA and negative control shRNA (sh-NC) were assembled by Shanghai GenePharma Co., Ltd. (Shanghai. China). The constructed lentivirus and target vectors were co-transfected into the 293 T cells while using the Lipofectamine 2000. After a period of incubation for 48 h, the cells were centrifuged. Following this, the supernatant was collected and filtrated and then the process of detection of viral titers was carried out. The cells were infected with control, sh-NC and sh-ERK viruses at the exponential phase. When the cardiomyocytes were at the logarithmic phase, they were trypsinized and triturated to 5 × 10^4^ cells/mL cell suspension. It was inoculated into a 6-well plate with 2 mL per well and was then incubated overnight at 37 °C. After 48 h of treatment, green fluorescent protein (GFP) expression efficiency was determined under a fluorescence microscope.

### Establishment of mouse PAH model

The mouse PAH model was established with the use of mice with gestational hypertension, which were produced by mating transgenic mice carrying the human renin (hRN) gene with transgenic mice carrying the human angiotensinogen (hANG) gene [12]. Healthy female C57BL/6J mice (4–6 weeks old; body weight: 21–25 g) were fed in accordance with the standard laboratory conditions (12 h light/dark cycle) and mated for pregnancy, which was used as a control group. The number of mice involved in the experiment was 70, which included 14 healthy C57BL/6J mice and 56 PAH mice. The PAH mice were intraperitoneally injected with LCZ696 (HY-18204A, MCE). The mice without treatment, PAH mice, and PAH mice treated with LCZ696 (n = 7) were used in order to count the survival rate at the 3rd, 4th, 5th, 6th, 7th, 8th, 9th, 10th, 11th and 12th weeks after injection, respectively. Besides, 7 mice in each group were euthanized with an injection of 3-time doses of 3% pentobarbital sodium (P3761, Sigma-Aldrich Chemical Company, St Louis MO, USA), and then the hearts of mice were frozen and sectioned for staining experiments. The lentiviruses stably transfected with sh-NC and sh-ERK were injected into the tail vein of PAH mice (n = 14). After a period of 1 week, LCZ696 (50 mg/kg, continuous injection once a day) was intraperitoneally injected into the mice treated with sh-NC and sh-ERK (n = 7). Next, the extent of ERK phosphorylation in the cardiac tissues of PAH mice was detected, and then the cardiac tissues of mice were marked through tissue staining and immunofluorescence staining.

### Hematoxylin-eosin (HE) staining

After the mice were euthanized using 3-time doses of 3% pentobarbital sodium (P3761, Sigma-Aldrich Chemical Company, St Louis MO, USA), they were perfused using 4 °C normal saline and 4% polyformaldehyde. The cardiac tissues of the mice were then collected, fixed overnight in 10% neutral formaldehyde solution, and finally embedded in paraffin. The tissues were then sectioned in a successive manner followed by HE staining. The sections were dewaxed, hydrated by gradient alcohol, washed under running water for 3 min, stained with hematoxylin for 8 min and eosin for 2 min, followed by routine dehydration, transparency, and resin sealing. At last, the cell morphology was analyzed under a high-power microscope.

### Masson’s trichrome staining

Cardiac tissue sections of mice were dewaxed into water, placed into Bouin’s solution, and then incubated in an incubator at 37 °C for 2 h. The sections were rinsed under running water until the color yellow disappeared. Afterwards, the sections were stained with Weigert’s iron hematoxylin for 10 min, differentiated with 1% hydrochloric acid alcohol, stained with fuchsin acid for 10 min, and then treated with 1% aqueous solution of phosphomolybdic acid for 5 min. Following this, the sections were counterstained with bright green for 5 min, treated with 1% glacial acetic acid for 1 min, dehydrated with gradient alcohol, and finally sealed with neutral balsam. The sections were subsequently examined under an optical microscope, and three fields were randomly selected for each section and the images were processed with the use of Image Pro Plus 6.0.

### Terminal deoxynucleotidyl transferase-mediated dUTP nick end-labeling (TUNEL) staining

To detect apoptotic cells, TUNEL assay was performed on paraffin-embedded heart sections of mice using the in situ Cell Death Detection Kit (Roche Diagnostics GmbH, Mannheim, Germany) according to the manufacturer’s instructions. For nuclear counterstaining, sections were subsequently stained with Hoechst 33258. The percentage of TUNEL-positive cells in 10 randomly chosen sections was determined using Win Roof software (Mitani Co., Ltd.). Fluorescence was visualized on a Leica DMR/XA fluorescence microscope (Fotoequipment: Leica DC 300F; Leica Microsystems GmbH, Wetzlar, Germany). The apoptotic positive cells were marked by tetramethylrhodamine in red and the normal cells were in blue. The apoptotic index was calculated by the ratio of the number of positive cells to the total number of cells.

Newborn mouse cardiomyocytes were rinsed with PBS three times and then fixed with 4% paraformaldehyde for 30 min. Then, 0.3% H_2_O_2_-formaldehyde solution at a ratio of 1:99 was prepared to further fix the samples for 30 min. The samples were then added with 0.3% Triton X-100 for 2 min. TUNEL reaction compounds were prepared in accordance to the instructions of the TUNEL Apoptosis Detection Kit (Promega, Fitchburg, WI, USA). The treatment groups were mixed with 50 μL reaction compounds and dUTP solution labeled with fluorescein while the NC group was added with 50 μL dUTP solution labeled with fluorescein. Afterwards, the cardiomyocytes were incubated at 37 °C for 60 min devoid of light, washed three times with PBS or Hank’s balanced salt solution (HBSS). The sections were sealed using anti-fluorescence quenching sealing solution and observed under a fluorescence microscope (Bio-Rad Laboratories, Hercules, CA, USA). Finally, the sections were detected at an excitation wavelength of 450 nm and an emission wavelength of 550 nm.

### RNA isolation and quantitation

Total RNA was extracted from the cells and tissues using a Trizol kit (Gibco BRL/Invitrogen, Carlsbad, CA, USA). The quality and concentration of the extracted RNA were determined with ultraviolet–visible spectrophotometry (ND-1000, Nanodrop, USA). PrimeScript RT Reagent kit (Takara, Dalian, Liaoning, China) was used to reversely transcribe the extracted RNA (400 ng). Subsequently, with complementary DNA (cDNA) as a template, reverse transcription quantitative polymerase chain reaction (RT-qPCR) was performed out in accordance with the manufacturer’s instructions of a SYBR^®^ Premix Ex Taq™ II (Tli RNaseH Plus) kit (Takara, Japan) in a Thermal Cycler Dice Real Time System amplify instrument (TP800, Takara, Tokyo, Japan). The primers were synthesized by Guangzhou Ribobio Co., Ltd. (Guangzhou, China), the sequences of which are shown in Table 1. With glyceraldehyde-3-phosphate dehydrogenase (GAPDH) serving as an internal reference, the fold changes were calculated by means of the relative quantification (2^−ΔΔCT^ method).

**Table 1:** Primer sequence for RT-qPCR

Genes	Primer sequences
ANP	F: 5′-GGGGGTAGGATTGACAGGAT-3′
	R: 5′-ACACACCACAAGGGCTTAGG-3′
β-MHC	F: 5′-CTACAGGCCTGGGCTTACC -3′
	R: 5′-TCTCCTTCTCAGACTTCCGC -3′
TIMP2	F: 5′-CCCGTAAGAAGGCTGACAGA-3′
	R: 5′-CCCTCCAGACCCACTACCAT -3′
Collagen I	F: 5′-ACAGGCGAACAAGGTGACAGAG -3′
	R: 5′-GCCAGGAGAACCAGCAGAGC-3′
Collage III	F: 5′-AGATGCTGGTGCTGAGAAGAAAC-3′
	R: 5′-GCTGGAAAGAAGTCTGAGGAAGG-3′
TGF-β	F: 5′-GGAAAGTGTTTCACCGCCAC-3′
	R: 5′-ACTGACACGTGACACTGGAC-3′
GAPDH	F: 5′-TTAGCACCCCTGGCCAAGG-3′
	R: 5′-CTTACTCCTTGGAGGCCATG-3′

*RT-qPCR* reverse transcription quantitative polymerase chain reaction, *ANP* atrial natriuretic peptide, *MHC* myosin heavy chain, *TIMP2* tissue inhibitor of metalloproteinase 2, *TGF-β* transforming growth factor-β, *GAPDH* glyceraldehyde-3-phosphate dehydrogenase, *F* forward, *R* reverse

### Western blot analysis

Left ventricular tissues or cardiomyocytes were rinsed with PBS, and lysed using Western cell lysis buffer (C0481, Sigma-Aldrich Chemical Company, St Louis, MO, USA) followed by incubation at 4 °C for 30 min. Cell lysate was then collected in 1.5 mL eppendorf (EP) tubes and centrifuged at 12,000×*g* at 4 °C for 15 min with the supernatant collected. A bicinchoninic acid (BCA) kit (Beyotime Biotechnology, Shanghai, China) was employed to measure the concentration of the total protein. The protein was separated using 10% sodium dodecyl sulfate–polyacrylamide gel electrophoresis (SDS-PAGE) and then transferred onto a polyvinylidene fluoride (PVDF) membrane (Millipore, Billerica, MA, USA), followed by sealing using 5% skimmed milk powder for 1 h. Then, the membrane was incubated overnight at 4 °C with the following diluted primary antibodies that were purchased from Abcam Inc. (Cambridge, UK): rabbit monoclonal antibody to ERK (ab32537, 1:1000), p-ERK (ab194776, 1:1000), caspase 3 (ab13847, 1:500), cleaved-caspase 3 (ab214430, 1:500), and GAPDH (ab181602, 1:10,000). Subsequently, the membrane was incubated with horseradish peroxidase (HRP)-labeled secondary antibody (ab99702, 1:1000, Abcam Inc., Cambridge, UK) for 1 h following 3 rinses with Tris-buffered saline Tween-20 (TBST). The immunocomplexes on the membrane were visualized using enhanced chemiluminescence (ECL) reagent (Baoman Biotechnology Co., Ltd, Shanghai, China) and band intensities were quantified using Image J gel imaging analysis software. The ratio of the gray value of the target band to GAPDH was representative of the relative protein expression.

### Immunofluorescence staining

Left ventricular tissues of mice were fixed in freshly prepared 2% paraformaldehyde (PFA) for 2 h, treated overnight with 10% sucrose solution, and then treated with 20% sucrose solution for 2 h. The frozen tissues were portioned into 4-μm-thick sections, and the octanol (OCT) was melted at room temperature to make the tissues attached the slides tightly. The tissues were subsequently penetrated and fixed in pre-cooled methanol at − 20 °C for 15 min. After sealed in 2% bovine serum albumin (BSA) and 5% goat serum for 60 min, the tissue sections were stained with isolectin B4 (Vector) for detection of capillary density and stained with a-actinin (Sigma-Aldrich Chemical Company, St Louis MO, USA) for detection of cardiomyocyte size of neonatal mice. After incubation at room temperature for 60 min devoid of light, the sections were stained with 1 mg/mL 4′,6-diamidino-2-phenylindole (DAPI) for nuclear staining, sealed with a fluorescent sealing agent, and then stored without any light at 4 °C (available within 1 week). All images were acquired with LEICA DC 500 camera on a microscope equipped with DMRA2 fluorescence optics (LEICA, Heidelberg, German) with 6 randomly chosen visual fields from each group.

### Statistical analysis

All experimental data were analyzed using SPSS 21.0 software (IBM Corp. Armonk, NY, USA). Firstly, the test of normality and homogeneity of variance exhibited that the measurement data conformed to the normality and homogeneity of variance. Measurement data were presented as mean ± standard deviation. Comparison among multiple groups was analyzed using one-way analysis of variance (ANOVA), followed by a Tukey post hoc test. Survival rate was calculated by the Kaplan–Meier method, and compared with a log-rank test. A *p* value of < 0.05 was considered to indicate statistical significance.

## Results

### LCZ696 attenuated cardiac injury in mice with PAH and suppresses the Ang II receptor pathway

**Fig. 1:**
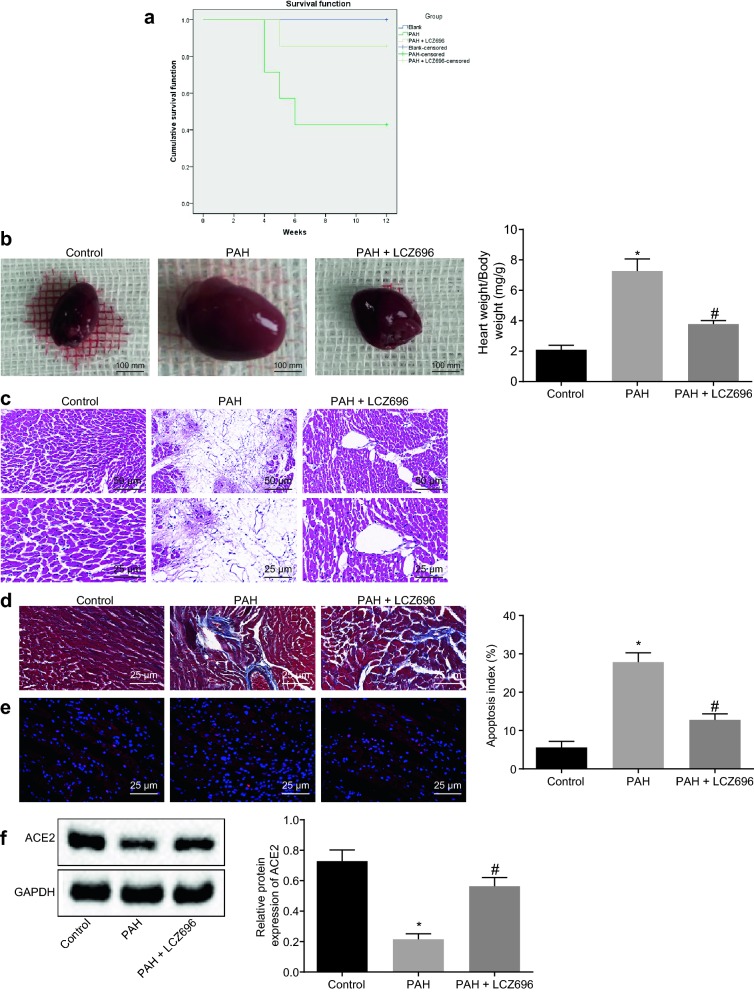
LCZ696 alleviates cardiac injury in PAH mice and represses the Ang II receptor pathway. PAH mice were either treated with or without LCZ696. **a** Effect of LCZ696 on survival rate of PAH mice detected by Kaplan–Meier method (N = 7). **b** Heart size (scale bar = 100 mm) and ratio of heart weight/body weight of mice. **c** HE staining analysis of cardiac tissues (upper panels: scale bar = 50 mm; lower panels: bar = 25 μm). **d** Cardiac fibrosis observed by Masson’s trichrome staining (scale bar = 25 μm). **e** Apoptosis of cardiomyocytes detected by TUNEL staining (scale bar = 25 μm). **f** Western blot analysis of ACE2 protein. N = 7. *p < 0.05 *vs.* normal mice; ^#^*p* < 0.05 *vs.* PAH mice. Measurement data (mean ± S.D.) among multiple groups were analyzed by one-way ANOVA, followed by Tukey post hoc test. Survival rate was calculated by the Kaplan–Meier method, and compared by a log-rank test

Firstly, we established the mouse PAH model, and then investigated the role of LCZ696 in PAH. After injection of LCZ696 (50 mg/kg), the Kaplan–Meier method was used to analyze the survival rate of PAH mice. The results displayed that the LCZ696 treatment improved the survival rate of PAH mice (Fig. 1a; *p* < 0.05), which suggest that LCZ696 may attenuate PAH. To further clarify the mechanism, we detected the heart size and weight of PAH mice. The results revealed that LCZ696 treatment decreased heart size and weight of PAH mice (Fig. 1b; *p* < 0.05). Moreover, HE staining analysis suggested that the necrosis of cardiomyocytes in PAH mice was critical. However, LCZ696 treatment reduced the necrosis of cardiomyocytes in PAH mice (Fig. 1c; *p* < 0.05). Subsequently, Masson’s trichrome staining was employed to further investigate the degree of cardiac fibrosis. As illustrated in Fig. 1d, PAH mice exhibited obvious cardiac and interstitial fibrosis while upon LCZ696 treatment, a decline was notable in cardiac and interstitial fibrosis (*p* < 0.05). Furthermore, TUNEL staining was performed to measure the apoptosis of cardiomyocytes, which demonstrated that PAH mice showed increased cardiomyocyte apoptosis while LCZ696 treatment decreased the apoptosis (Fig. 1e; *p* < 0.05). Finally, western blot analysis indicated that a relationship eisted and that LCZ696 increased the expression of ACE2 (Fig. 1f; *p* < 0.05).

The above results demonstrate that LCZ696 could alleviate the cardiac remodeling in PAH mice through the inhibition of the Ang II receptor pathway.

### LCZ696 attenuated cardiac hypertrophy and fibrosis induced by Ang II through inhibition of ACE2 expression

**Fig. 2:**
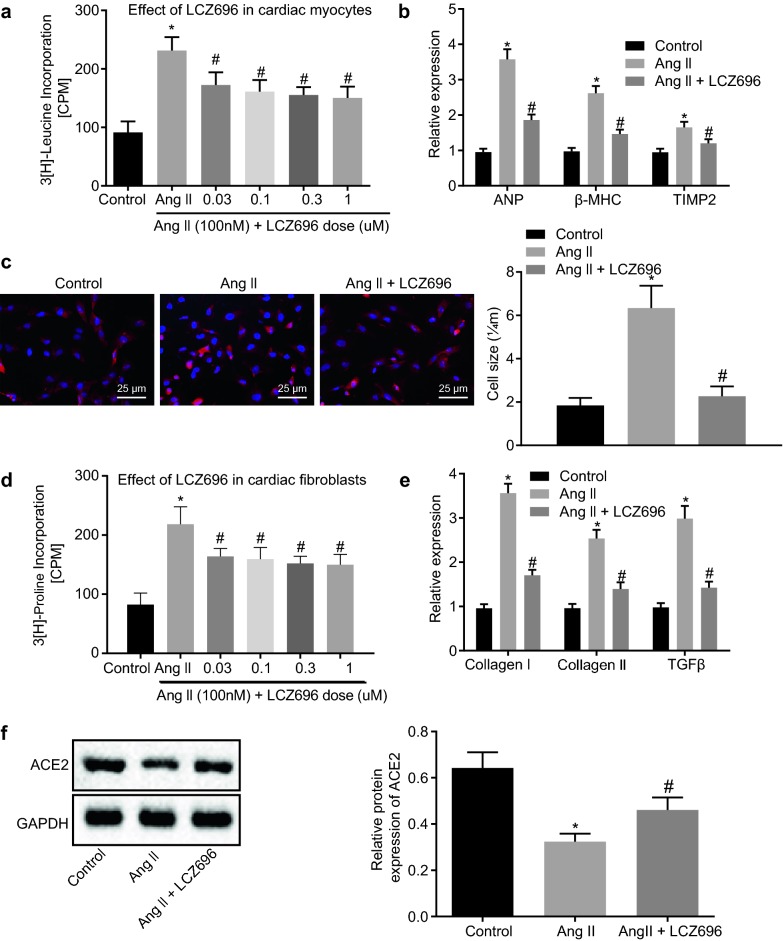
LCZ696 alleviates Ang II-induced cardiac hypertrophy and fibrosis. Extracted cardiomyocytes were treated with Ang II (100 nM) alone or with LCZ696 (1.0 μM), or without any treatment. **a** Cardiac hypertrophy condition detected by 3[H]-leucine incorporation methodology. **b** mRNA expression of ANP, βMHC and TIMP2 in cardiomyocytes detected by RT-qPCR. **c** Size of cardiomyocytes detected by a-actinin immunofluorescence staining and percentage of cardiomyocyte size (scale bar = 25 μm). **d** Cardiac fibrosis assessed by 3[H]-proline incorporation methodology. **e** mRNA expression of collagen I, collagen III and TGF-β detected by RT-qPCR. F, Western blot analysis of ACE2 protein in cardiomyocytes. *p < 0.05 *vs.* cardiomyocytes without treatment; ^#^*p* < 0.05 *vs.* cardiomyocytes treated with Ang II. Measurement data (mean ± S.D.) among multiple groups was analyzed by one-way ANOVA, followed by Tukey post hoc test. The experiment was conducted 3 times independently

To further investigate how LCZ696 regulated cardiac remodeling in vitro, we treated cardiomyocytes and cardiac fibroblasts with Ang II and different concentrations (0.03/0.1/0.3/1.0 μM) of LCZ696. 3[H]-leucine incorporation methodology was subsequently used in order to detect the cardiac hypertrophy. The results portrayed that Ang II treatment increased the 3[H]-leucine incorporation, while LCZ696 treatment decreased the 3[H]-leucine incorporation, and higher concentration of LCZ696 decreased the 3[H]-leucine incorporation more evidently (Fig. 2a; *p* < 0.05). The results suggest that LCZ696 treatment can decrease Ang II-induced cardiac hypertrophy. Next, RT-qPCR was performed in order to verify the expression of markers of cardiac hypertrophy (ANP, βMHC and TIMP2). The data showed that 100 nM Ang II increased the expression of ANP, βMHC and TIMP2, while 1.0 μM LCZ696 decreased the expression of ANP, βMHC and TIMP2 (Fig. 2b; *p* < 0.05). Immunofluorescence staining analyses of cardiac hypertrophy and cardiomyocyte size demonstrated that 100 nM Ang II increased the cardiomyocyte size, while 1.0 μM LCZ696 decreased the cardiomyocyte size (Fig. 2c; *p* < 0.05). In addition, the same trend of 3[H]-proline incorporation was discovered for the cardiac fibrosis as for cardiac fibroblast hypertrophy (Fig. 2d; *p* < 0.05), suggesting that LCZ696 treatment could decrease Ang II-induced cardiac fibrosis. Similarly, RT-qPCR was conducted to detect the expression of collagen I, collagen III and TGF-β. Expectedly, 100 nM Ang II treatment elevated the expression of collagen I, collagen III and TGF-β, while a decline was observed following 1.0 μM LCZ696 treatment (Fig. 2e; *p* < 0.05). Subsequent western blot analysis suggested that 100 nM Ang II treatment reduced the protein expression of ACE2 in cardiomyocytes, while 1.0 μM LCZ696 treatment fostered the protein expression of ACE2 (Fig. 2f; *p* < 0.05).

Taken together, the aforementioned data suggest that LCZ696 treatment can alleviate the cardiac hypertrophy and fibrosis via inhibition of the Ang II receptor pathway.

### LCZ696 restrained apoptosis of cardiomyocytes induced by Ang II

**Fig. 3:**
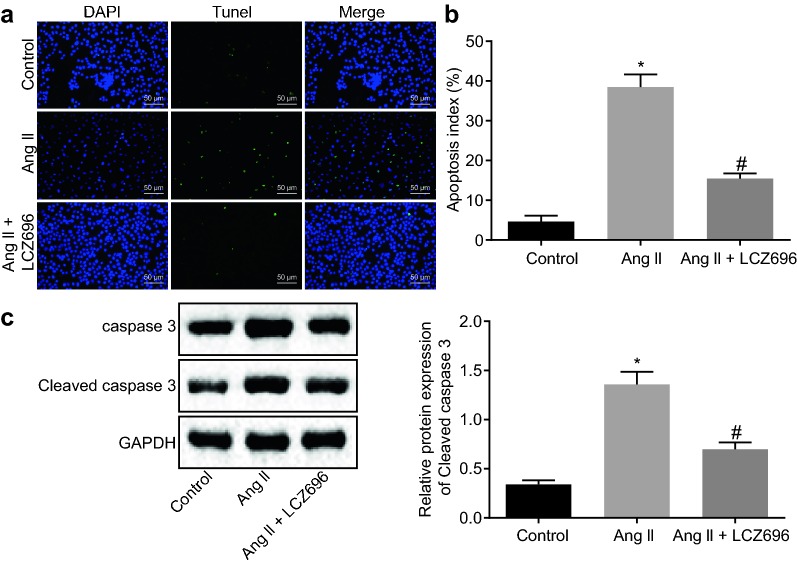
LCZ696 impedes Ang II-induced apoptosis of cardiomyocytes. Extracted cardiomyocytes were treated with Ang II (100 nM) alone or with LCZ696 (1.0 μM), or without any treatment. **a** Apoptosis of cardiomyocytes treated with LCZ696 in vitro detected by TUNEL staining (scale bar = 25 μm). **b** Quantitative analysis for AI of cardiomyocytes. **c** Western blot analysis of caspase 3 and cleaved-caspase 3 proteins in cardiomyocytes treated with LCZ696 or Ang II. *p < 0.05 *vs.* cardiomyocytes without treatment; ^#^*p* < 0.05 *vs.* cardiomyocytes treated with Ang II. Measurement data (mean ± S.D.) among multiple groups was analyzed by one-way ANOVA, followed by Tukey post hoc test. The experiment was conducted 3 times independently

Furthermore, in order to explore the effect of LCZ696 on the biological function of cardiomyocytes treated with Ang II, the apoptosis of cardiomyocytes treated with LCZ696 was measured with the use of TUNEL staining. The results depicted in Fig. 3a, b displayed that 100 nM Ang II treatment increased AI while the AI gradually decreased upon 1.0 μM LCZ696 treatment (*p* < 0.05). Afterwards, western blot analysis was employed to verify the expression of apoptosis-related proteins: caspase 3 and cleaved-caspase 3. The results exhibited that Ang II treatment increased expression and activity of caspase 3 and cleaved-caspase 3 in cardiomyocytes, while treatment with LCZ696 reversed the trend (Fig. 3c; *p* < 0.05).

Therefore, it can be asserted that LCZ696 has potential to decrease Ang II-induced apoptosis of cardiomyocytes.

### LCZ696 diminished cardiac hypertrophy and fibrosis induced by Ang II through inhibition of ERK phosphorylation

**Fig. 4:**
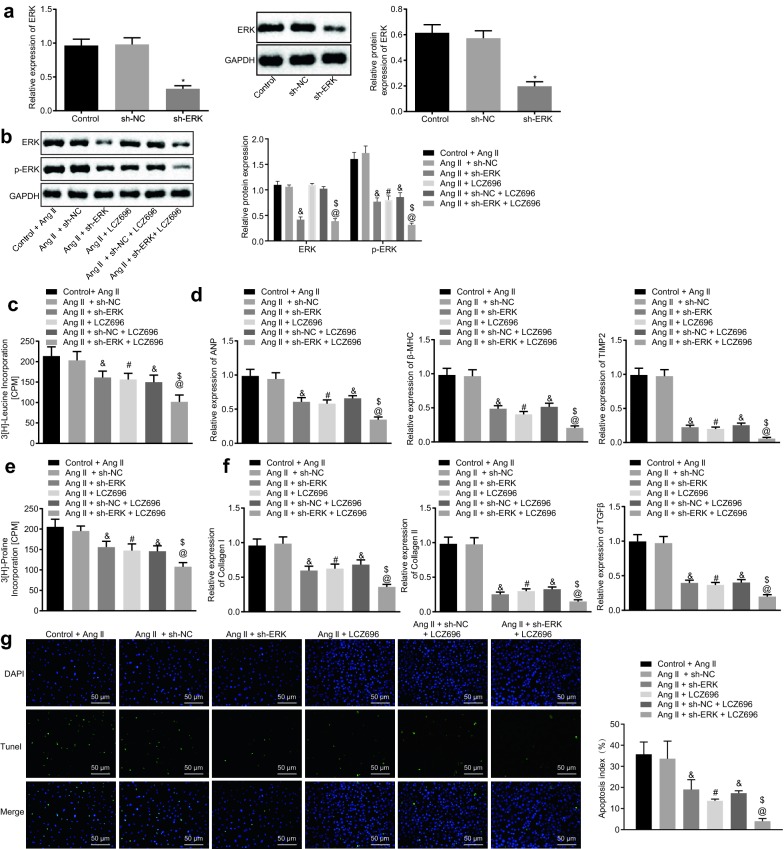
LCZ696 diminishes cardiac hypertrophy and fibrosis induced by Ang II via suppression of the ERK phosphorylation. Cardiomyocytes were initially treated with either sh-NC or sh-ERK, or without any treatment. **a** mRNA and protein expression of ERK detected by RT-qPCR and western blot analysis. **b** Western blot analysis of the extent of ERK phosphorylation in cardiomyocytes upon different treatment. **c** Cardiac hypertrophy condition assessed by 3[H]-leucine incorporation methodology. **d** mRNA expression of ANP, βMHC and TIMP2 in cardiomyocytes detected by RT-qPCR. E, Cardiomyocyte fibrosis assessed by 3[H]-proline incorporation methodology. F, mRNA expression of collagen I, collagen III and TGF-β probed by RT-qPCR. G, Apoptosis of cardiomyocytes treated with LCZ696 as probed by TUNEL staining (scale bar = 25 μm). *p < 0.05 *vs.* cardiomyocytes without treatment; ^#^*p* < 0.05 *vs.* cardiomyocytes treated with control + Ang II; ^&^*p* < 0.05 *vs.* cardiomyocytes treated with Ang II + sh-NC; ^@^*p* < 0.05 *vs.* cardiomyocytes treated with Ang II + sh-ERK; ^$^*p* < 0.05 *vs.* cardiomyocytes treated with Ang II + sh-NC + LCZ696. Measurement data (mean ± S.D.) among multiple groups was analyzed by one-way ANOVA, followed by Tukey post hoc test. The experiment was conducted 3 times independently

To further verify whether the functionality of LCZ696 in cardiac hypertrophy and fibrosis was correlated with the ERK signaling pathway, we silenced the ERK gene in cardiomyocytes and cardiac fibroblasts treated with Ang II. Then RT-qPCR and western blot analysis were performed in order to determine the expression of ERK in cells treated with silencing ERK, which revealed that mRNA and protein expression of ERK was decreased in cells treated with sh-ERK, suggesting a success of ERK silencing transfection (Fig. 4a; *p* < 0.05). Next, we conducted western blot analysis to evaluate the extent of ERK phosphorylation in cells treated Ang II + LCZ696 and Ang II + sh-ERK + LCZ696. As portrayed in Fig. 4b, cells treated with Ang II showed upregulated extent ERK of phosphorylation, which was blocked upon Ang II + sh-ERK treatment (*p* < 0.05). The cardiac hypertrophy was subsequently detected by the 3[H]-leucine incorporation experiment. It was noted that Ang II-treated cells showed increased 3[H]-leucine incorporation while 3[H]-leucine incorporation was reduced in AngII + sh-ERK-treated cells (Fig. 4c; *p* < 0.05). Meanwhile, the expression levels of markers of cardiac hypertrophy (ANP, βMHC and TIMP2) were quantified through the performance of RT-qPCR. The results were coincided with those of 3[H]-leucine incorporation (Fig. 4d; *p* < 0.05). Similar experiments were conducted to quantify the effects of LCZ696 on cardiac fibroblasts. It was found that the cardiac fibroblasts treated with Ang II + sh-NC + LCZ696 showed decreased 3[H]-leucine incorporation (*p* < 0.05). In comparison with cardiac fibroblasts treated with Ang II + sh-ERK, cardiac fibroblasts treated with Ang II + sh-ERK + LCZ696 showed decreased 3[H]-proline incorporation (Fig. 4e; *p* < 0.05). Similarly, RT-qPCR was used to detect the expression of markers for cardiac fibroblasts (collagen I, collagen III and TGF-β), and the results were consistent with those of 3[H]-proline incorporation (Fig. 4f). Furthermore, TUNEL staining suggested that the cardiomyocytes treated with Ang II + sh-NC + LCZ696 showed decreased apoptosis of cardiomyocytes (*p* < 0.05). When compared with cardiomyocytes treated with Ang II + sh-ERK, cardiomyocytes treated with Ang II + sh-ERK + LCZ696 exhibited decreased apoptosis of cardiomyocytes (Fig. 4g; *p* < 0.05).

The above results demonstrate that LCZ696 inhibits the phosphorylation of ERK in cardiomyocytes and cardiac fibroblasts, thereby reducing Ang II-induced cardiac hypertrophy, fibrosis, and apoptosis.

### LCZ696 alleviated cardiac remodeling of PAH mice through inhibition of the ERK signaling pathway

**Fig. 5:**
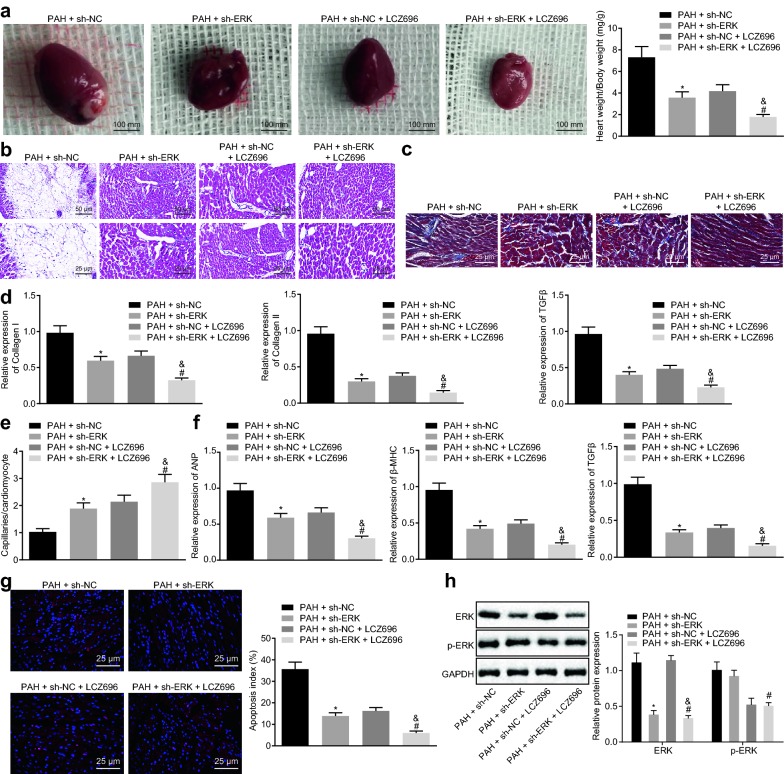
LCZ696 reduces cardiac remodeling through inhibiting the ERK signaling pathway. PAH mice were treated with sh-ERK with sh-NC as the control, or sh-ERK in the presence of LCZ696 with sh-NC in the presence of LCZ696 or control. **a** Heart size (scale bar = 100 mm) and ratio of heart weight/body weight in PAH mice. **b** HE staining of ventricular transection of heart (upper panels: scale bar = 50 mm; lower panels: scale bar = 25 μm). **c** Cardiomyocyte fibrosis observed by Masson’s trichrome staining (scale bar = 25 μm). **d** mRNA expression of collagen I, collagen III and TGF-β detected by RT-qPCR. **e** Isolectin B4 immunofluorescence of the capillary in ventricular tissues (blood vessel: yellow; cell membrane: red; nucleus: blue). **f** mRNA expression of ANP, βMHC and TIMP2 in cardiomyocytes detected by RT-qPCR. **g** Apoptosis of cardiomyocytes treated with LCZ696 detected by TUNEL staining (scale bar = 25 μm). **h** Western blot analysis of ERK protein and extent of ERK phosphorylation. *p < 0.05 *vs.* PAH mice treated with sh-NC; ^#^*p* < 0.05 *vs.* PAH mice treated with sh-ERK; ^&^*p* < 0.05 *vs.* PAH mice treated with sh-NC + LCZ696. N = 7. Measurement data (mean ± S.D.) among multiple groups was analyzed by one-way ANOVA, followed by Tukey post hoc test

Finally, to verify the role of LCZ696 in PAH mice through the regulation of ERK signaling pathway, we conducted in vivo experiments in which PAH mice were injected with lentiviral vector stably transfected with either sh-ERK or LCZ696. Initially, the heart size and weight of PAH mice was measured, and the data indicated that PAH mice treated with sh-ERK showed decreased heart weight and size (*p* < 0.05). Furthermore, when compared with the PAH mice treated with sh-ERK or sh-NC + LCZ696, the PAH mice treated with sh-ERK + LCZ696 also showed decreased heart weight and size (Fig. 5a; *p* < 0.05). HE staining was then employed to examine the degree of cardiac injury in PAH mice. As depicted in Fig. 5b, PAH mice treated with sh-ERK showed decreased degree of cardiomyocyte necrosis (*p* < 0.05). Similarly, in comparison to the PAH mice treated with sh-ERK or sh-NC + LCZ696, the PAH mice treated with sh-ERK + LCZ696 also showed decreased cardiomyocyte necrosis (*p* < 0.05). Masson’s trichrome staining revealed that PAH mice upon sh-ERK treatment presented with decreased cardiac and interstitial fibrosis (*p* < 0.05). In comparison with the PAH mice treated with sh-ERK or sh-NC + LCZ696, the PAH mice treated with sh-ERK + LCZ696 exhibited decreased cardiac and interstitial fibrosis (Fig. 5c; *p* < 0.05). Detection of the expression levels of markers of cardiac fibroblasts (collagen I, collagen III and TGF-β) by RT-qPCR conveyed a similar decreasing trend as the Masson’s trichrome staining (Fig. 5d; *p* < 0.05) under the same conditions. Furthermore, isolectin B4 immunofluorescence was utilized to measure the capillary number in ventricular tissues and the ratio of capillary to cardiomyocyte size was analyzed statistically. The results displayed increased capillary number in ventricular tissues and ratio of capillary to the cardiomyocyte size in PAH mice treated with sh-ERK (*p* < 0.05). A similar result was noted in PAH mice treated with sh-ERK + LCZ696 upon comparing to the PAH mice treated with sh-ERK or sh-NC + LCZ696 (Fig. 5e; *p* < 0.05). The mRNA expression of cardiac hypertrophy markers (ANP, βMHC and TIMP2) was detected by RT-qPCR, and the results demonstrated that silenced ERK decreased the expression of ANP, βMHC and TIMP2 (*p* < 0.05). The expression of ANP, βMHC and TIMP2 was further reduced by the addition of LCZ696, which was lower than that in the corresponding control (Fig. 5f; *p* < 0.05). TUNEL staining illustrated that silenced ERK diminished the apoptosis of cardiomyocytes, and LCZ696 treatment further reduced the apoptosis of cardiomyocytes (Fig. 5g; *p* < 0.05). Subsequent western blot analysis supported that silenced ERK disrupted ERK expression and extent of ERK phosphorylation, and LCZ696 treatment could diminish the ERK expression and extent of ERK phosphorylation (Fig. 5h; *p* < 0.05).

The results further verify that LCZ696 could inhibit the ERK signaling pathway to prevent cardiac remodeling.

## Discussion

Ang-converting enzyme inhibitors have been extensively reported to act as the peripartum cardiomyopathy-targeted therapies [13]. LCZ696 is commonly known as an Ang II receptor inhibitor and has been proven to be an effective drug for the prevention of hypertension and cardiovascular diseases [6]. However, its specific role and the underlining molecular mechanism in PAH is yet to be investigated. Therefore, the objective of this present study was to elucidate the mechanisms by which LCZ696 was implicated in the process of cardiac remodeling in mice with PAH. The obtained findings suggested that LCZ696 could potentially alleviate cardiac hypertrophy and fibrosis, consequently ameliorating cardiac remodeling through inhibition of the ERK signaling pathway in PAH mice.

Initially, it was found that LCZ696 could attenuate cardiac injury in PAH mice. LCZ696, a first-in-class inhibitor of Ang II receptor and neprilysin, has the potency to lower blood pressure effectively and be well-tolerated in patients with hypertension [14]. A study demonstrated that the patients with hypertension can benefit from routine management with Ang-converting enzyme inhibitors or Ang receptor blockers [15]. A study supported the potential benefit of LCZ696 which weakens left ventricular remodeling following experimental cardiac infarction by repressing hypertrophy and fibrosis in cardiomyocytes, [16], which coincided with our results.

Subsequently, it was also found that LCZ696 could inhibit cardiac hypertrophy, fibrosis and apoptosis induced by Ang II in mouse model with PAH. Moreover, LCZ696 could inhibit the expression of ANP, βMHC and TIMP2, collagen I, collagen III and TGF-β. Ang II, a vasopressor hormone, functions in maintaining normal blood pressure and pathogenesis of cardiovascular diseases, and its overproduction forces the occurrence of hypertension in late pregnancy of mice with PAH [17]. A previous study has demonstrated the ability of Ang II to induce high ANP and βMHC levels in cardiomyocytes in cardiac hypertrophy [18]. TIMP2 and TIMP3 are regulators of cardiac remodeling, hypertrophy, and fibrosis in heart disease and can be induced by Ang II [19]. Ang II was also able to induce collagen gene transcription with the involvement of the TGF-β superfamily in cardiac fibroblasts [20]. Consistent with our study, it has been displayed that expression of hypertrophic markers (ANP and βMHC) was diminished after LCZ696 treatment in comparison with that of perindopril, and LCZ696 could also contribute to the decline in cardiac TIMP2 expression by decreasing collagen I expression in diabetic mice [21]. Following cardiac reperfusion injury, the fibrotic area was analyzed to be decreased and TGF-β expression in the left ventricle is inhibited in diabetic mice treated with LCZ696 for 4 weeks [22]. Furthermore, increased cardiomyocyte apoptosis could be significantly attenuated by LCZ696 in vitro [11]. Therefore, our work confirms the previously established role of Ang II blocker LCZ696, which makes LCZ696 a potential target for the treatment of patients with PAH.

Finally, we discovered that LCZ696 ameliorated the cardiac remodeling in PAH mice via inhibition of the ERK signaling pathway. Although there was no supporting evidence for the direct correlation of LCZ696 and the ERK signaling pathway, the ERK signaling pathway has been frequently understood to be correlated with Ang II in different studies. For instance, the blockade of ERK has the ability to efficaciously impair Ang II-induced cardiac hypertrophy and apoptosis through the inhibition of the insulin-like growth factor II receptor (IGF-IIR) signaling pathway [23]. In diabetic rats, the inhibited ERK signaling pathway was noted to inhibit cardiac hypertrophy and fibrosis [24]. The inhibition of ERK has been documented to protect against hyperglycaemia-induced cardiomyocyte apoptosis [25]. Furthermore, Ang II could induce the activation of ERK1/2 proteins along with the expression of ANP and βMHC in cardiomyocytes, which could be reversed by puerarin (Pue), an isoflavone derived from Kudzu roots, and then Ang II-induced cardiac hypertrophy was alleviated [26]. Moreover, high glucose and Ang-II directly affect the production of extracellular vesicles derived from endothelial cells, and aggravate endothelial dysfunction by upregulating ERK1/2 signaling pathway in mouse aorta [27]. Therefore, this study initially demonstrated that LCZ696 is also an inhibitor of the ERK signaling pathway so as to attenuate PAH.

## Conclusion

**Fig. 6:**
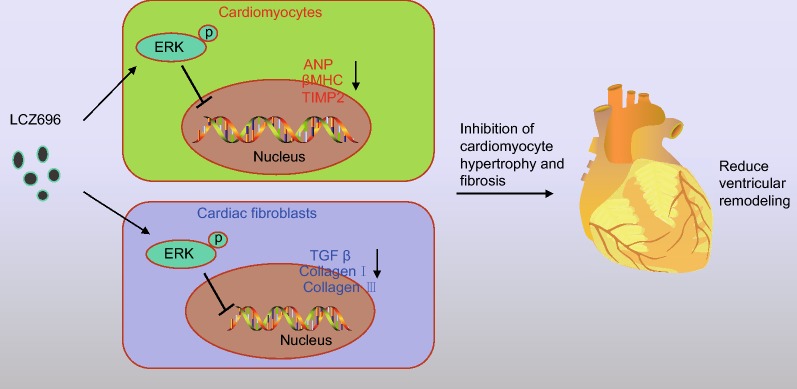
Potential molecular mechanisms of Ang II receptor inhibitor LCZ696 in PAH by regulating the ERK signaling pathway. In vivo and in vitro, LCZ676 reduces the expression of ANP, βMHC, TIMP2, Collagen I, Collagen III and TGF-β in cardiomyocytes by inhibiting ERK phosphorylation, thus diminishing cardiac hypertrophy and fibrosis, along with cardiomyocyte apoptosis, which ultimately attenuates cardiac remodeling in mice with PAH

In conclusion, this current study established that LCZ696, as an Ang II receptor inhibitor, attenuates PAH in mice through the inhibition of the activation of the ERK signaling pathway (Fig. 6). LCZ696-targeted therapy proves to present a promising potential as a novel therapeutic approach in the treatment of PAH. However, further investigation is necessary to shed light on the effect that LCZ696 has on embryonic development in PAH animal model.

## Data Availability

The datasets generated/analysed during the current study are available.

## Abbreviations

AI: apoptotic index
Ang: angiotensin
ANOVA: analysis of variance
ANP: atrial natriuretic peptide
BCA: bicinchoninic acid
BSA: bovine serum albumin
cDNA: complementary DNA
DAB: diaminobenzidine
DAPI: 4′,6-diamidino-2-phenylindole
DMEM: Dulbecco’s modified Eagle’s medium
DMSO: dimethyl sulfoxide
ECL: enhanced chemiluminescence
EP: eppendorf
ERK: extracellular signal-regulated kinase
FBS: fetal bovine serum
GAPDH: glyceraldehyde-3-phosphate dehydrogenase
GFP: green fluorescent protein
hANG: human angiotensinogen
HBSS: Hank’s balanced salt solution
HE: hematoxylin–eosin
hRN: human renin
HRP: horseradish peroxidase
MAPK: mitogen activated-protein kinase
MHC: β-myosin heavy chain
NC: negative control
OCT: octanol
PAH: pregnancy-associated hypertension
PBS: phosphate-buffered saline
POD: peroxidase
PVDF: polyvinylidene fluoride
shRNA: short hairpin RNA
RT-qPCR: reverse transcription quantitative polymerase chain reaction
SDS-PAGE: sodium dodecyl sulfate–polyacrylamide gel electrophoresis
TBST: tris-buffered saline Tween-20
TGF: transforming growth factor
TIMP2: tissue inhibitor of metalloproteinase 2
TUNEL: terminal deoxynucleotidyl transferase-mediated dUTP nick end-labeling
