# Correction to: Angiotensin II receptor blocker LCZ696 attenuates cardiac remodeling through the inhibition of the ERK signaling pathway in mice with pregnancy‑associated cardiomyopathy

**DOI:** 10.1186/s13578-020-00419-x

**Published:** 2020-05-04

**Authors:** Yi Wang, Zhiheng Guo, Yongmei Gao, Ping Liang, Yanhong Shan, Jin He

**Affiliations:** Department of Obstetrics, The First Hospital of Jilin University, No. 71, Xinmin Street, Changchun, 130021 Jilin People’s Republic of China

## Correction to: Cell BioSci (2019) 9:86 10.1186/s13578-019-0348-1

Following publication of the original article [1], the authors identified an error in Figs. 1b, 5b. The corrected figures are given below.

**Fig. 1:**
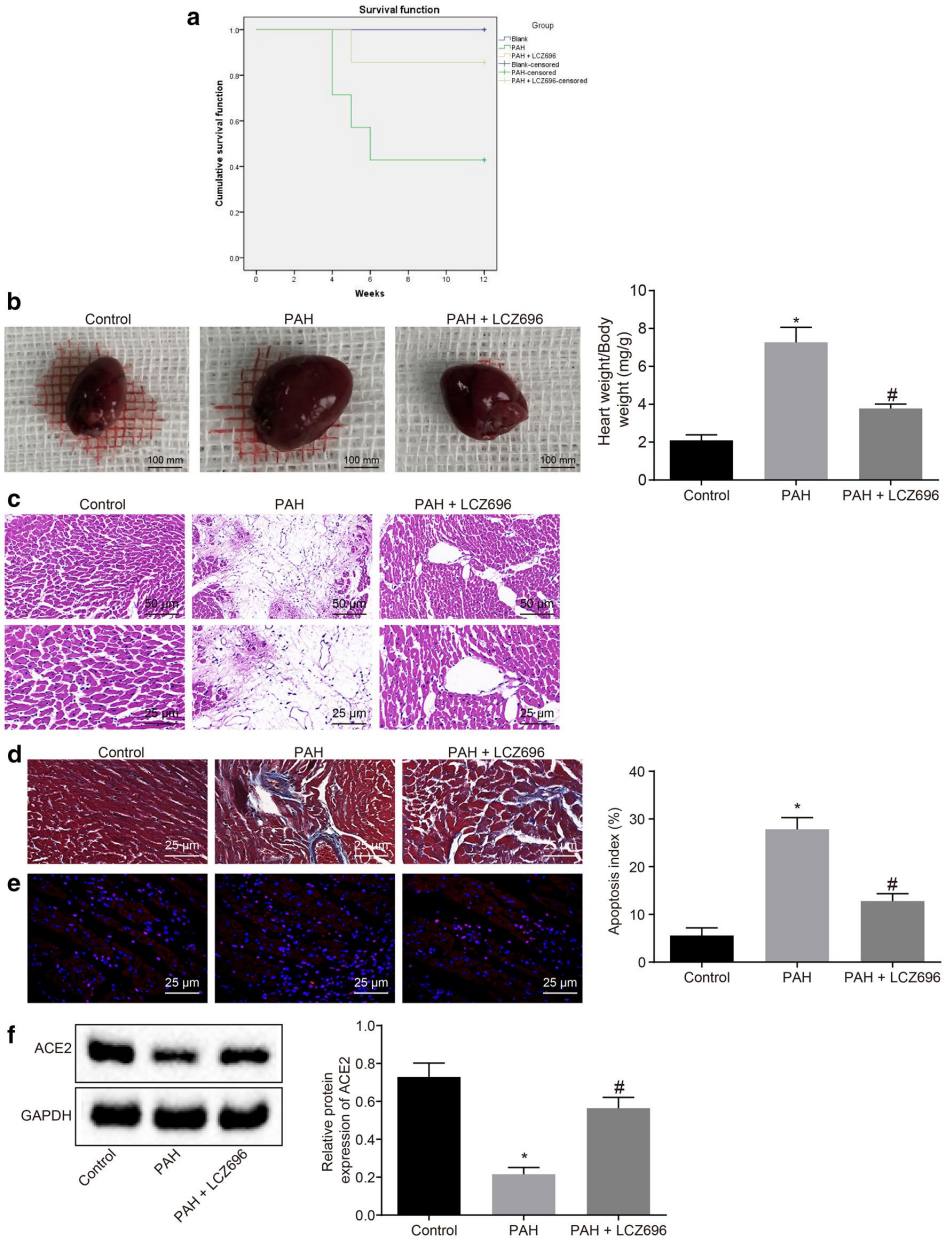
LCZ696 alleviates cardiac injury in PAH mice and represses the Ang II receptor pathway. PAH mice were either treated with or without LCZ696. **a** Effect of LCZ696 on survival rate of PAH mice detected by Kaplan–Meier method (N = 7). **b** Heart size (scale bar = 100 mm) and ratio of heart weight/body weight of mice. **c** HE staining analysis of cardiac tissues (upper panels: scale bar = 50 mm; lower panels: bar = 25 μm). **d** Cardiac fibrosis observed by Masson’s trichrome staining (scale bar = 25 μm). **e** Apoptosis of cardiomyocytes detected by TUNEL staining (scale bar = 25 μm). **f** Western blot analysis of ACE2 protein. N = 7. *p < 0.05 vs. normal mice; ^#^*p* < 0.05 vs. PAH mice. Measurement data (mean ± S.D.) among multiple groups were analyzed by one-way ANOVA, followed by Tukey post hoc test. Survival rate was calculated by the Kaplan–Meier method, and compared by a log-rank test

**Fig. 5:**
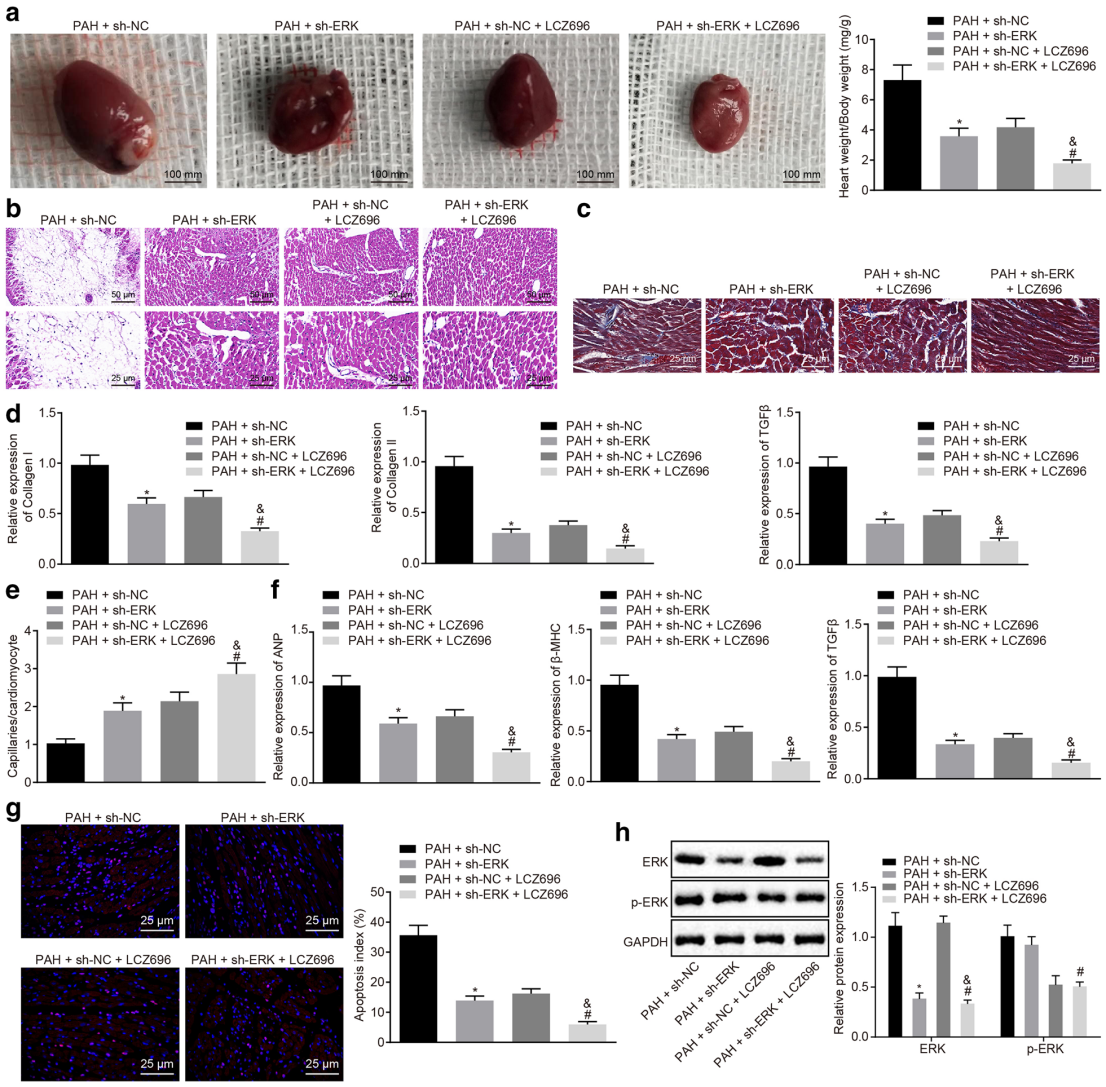
LCZ696 reduces cardiac remodeling through inhibiting the ERK signaling pathway. PAH mice were treated with sh-ERK with sh-NC as the control, or sh-ERK in the presence of LCZ696 with sh-NC in the presence of LCZ696 or control. **a** Heart size (scale bar = 100 mm) and ratio of heart weight/body weight in PAH mice. **b** HE staining of ventricular transection of heart (upper panels: scale bar = 50 mm; lower panels: scale bar = 25 μm). **c** Cardiomyocyte fibrosis observed by Masson’s trichrome staining (scale bar = 25 μm). **d** mRNA expression of collagen I, collagen III and TGF-β detected by RT-qPCR. **e** Isolectin B4 immunofluorescence of the capillary in ventricular tissues (blood vessel: yellow; cell membrane: red; nucleus: blue). **f** mRNA expression of ANP, βMHC and TIMP2 in cardiomyocytes detected by RT-qPCR. **g** Apoptosis of cardiomyocytes treated with LCZ696 detected by TUNEL staining (scale bar = 25 μm). **h** Western blot analysis of ERK protein and extent of ERK phosphorylation. *p < 0.05 vs. PAH mice treated with sh-NC; ^#^*p* < 0.05 vs. PAH mice treated with sh-ERK; ^&^*p* < 0.05 vs. PAH mice treated with sh-NC + LCZ696. N = 7. Measurement data (mean ± S.D.) among multiple groups was analyzed by one-way ANOVA, followed by Tukey post hoc test
